# Whitening: is Omnichroma universal composite unchanging? Spectrophotometric evaluation

**DOI:** 10.4317/jced.62204

**Published:** 2024-12-01

**Authors:** Francesca Zotti, Francesca Ferrari, Luciano Malchiodi, Carlotta Dorigatti, Francesca Pilati, Giorgia Lanzaretti, Nicoletta Zerman

**Affiliations:** 1 Department of Surgical Sciences, Pediatrics and Gynecology, University of Verona, P.le L.A.Scuro, 10, 37134 Verona, Italy; 2 Private Practice Verona, 37060, Italy; 3 Private Practice Trento, 38121, Italy; 4 Private Practice Vicenza, 36030, Italy

## Abstract

**Background:**

This study aimed to evaluate the color stability of Class V anterior restorations with universal composite after professional bleaching using a spectrophotometer.

**Material and Methods:**

Class V cavities were prepared and restored with universal composite in twenty-eight extracted anterior teeth. One week after restoration, color analysis was performed using the spectrophotometer. In-office bleaching was performed. Color analysis was performed 24 hours, 72 hours and 30 days after bleaching. The parameters evaluated were L* values of each tooth and ΔE values between tooth and restoration at different timepoints. Data were analyzed using a statistical software. A P-value ≤0.05 was used to indicate statistical significance of the results.

**Results:**

There was a non-statistically significant but noticeable difference between the L* values in term of tooth bleaching effect. Non-statistically significant differences were found between the L* values and the ΔE values at different timepoints when evaluating the chromatic difference between the tooth surface and the restoration. The ΔE value one week after the restoration is higher than the ΔE values at the following timepoints, therefore the color difference between the restoration and the adjacent tooth decreases with time after bleaching.

**Conclusions:**

Universal Composite was found to be able to match the color of the surrounding tooth even after the bleaching procedure.

** Key words:**Omnichroma, composite, color, spectrophotometer, whitening.

## Introduction

In recent years, the aesthetic outcome has assumed an increasingly vital role, impacting both patients and clinicians. Consequently, there has been a heightened focus on research, encompassing procedural methodologies and the exploration of materials (1-4), aimed at attaining outcomes that are both enduring and aesthetically pleasing (5). In the field of dentistry, resin-based composite materials have garnered widespread acclaim (6). The significant development in the composition of these materials throughout history is unquestionable. Manufacturers have fiercely competed to enhance and rectify any structural weaknesses that could result in clinical challenges, including ensuring seamless structural and optical integration between the composite restoration and the natural tooth structure as well as neighbouring teeth (7). Achieving this necessitates the use of composite resin with varying opacities and shades to match the color subtleties of the teeth, a process that can be time-intensive for both the dental practitioner and the patient (8). Moreover, the clinical effectiveness of dental composites relies on their physical, chemical, and mechanical attributes, which are substantially influenced by both the oral environment and the inherent properties of the resin material (9).

Recently, universal composite materials have made their debut in the market, aiming to streamline the inventory of composite shades, reduce waste, minimize chair-side time, eliminate the need for shade selection, and decrease reliance on shade-matching procedures. Developers claim that the primary advantage of these composites lies in their enhanced Color Adjustment Potential (CAP), a property defined as the “interaction between the physical and perceptual aspects of blending” (10). These materials possess universal opacity and are available in a limited range of VITA shades. Developers recommend their use in a single shade increment that may potentially harmonize with various tooth colors (10). The resin matrix of these composites primarily comprises Bis-GMA (bisphenol-A glycidyl dimethacrylate), combined in various proportions with short-chain monomers like TEGDMA (triethylene glycol-dimethacrylate), UDMA (urethane dimethacrylate), Bis-EMA (Bisphenol A polyethylene glycol diether dimethacrylate), and other monomers. The fillers consist of glass, silica, or zirconia, with varying filler contents and shapes.

A recent addition to the universal resin-based composites is Omnichroma from Tokuyama Dental Corp, Japan. It offers dental practitioners a convenient solution to a common challenge, which is selecting the appropriate shade. According to the manufacturer, Omnichroma is a universal shade composite with advanced chromatic technology that governs its optical characteristics. This method ensures an accurate reflection of a specific wavelength within the natural tooth color spectrum (11). Consequently, it can match all VITA classical A1-D1 shades with a single universal shade. Omnichroma consists of equal parts of zirconium dioxide (ZrO2) mixed with supra-nanospherical silicon dioxide (SiO2) filler particles measuring 260 nm in size, in addition to round-shaped composite filler particles with similar characteristics. According to the manufacturer, Omnichroma becomes more translucent after polymerization, with a refractive index of 1.47 before and 1.52 after polymerization. This aligns with prior research that identified a strong correlation between the translucency parameter and the blending effect associated with color adaptation (11). Once placed in the cavity preparation, this shadeless composite promptly adopts the color of the underlying and surrounding dentin and enamel, saving both time for the dental practitioner and the patient and eliminating the need for shade selection.

Hydrogen peroxide (HP), available in various concentrations ranging from 3% to 40%, decomposes into hydroxy-free radicals when exposed to light or heat and is a widely used bleaching agent to break down double bonds or ring structures present in stains (12). The effectiveness of bleaching primarily depends on the bleaching protocol used, including HP concentration, bleaching duration, and the composition of restorative materials, such as the structure of the resin matrix as well as the properties of the filler particles (13)-(14). Dental bleaching can be performed on both vital and nonvital teeth (12). There are two main techniques for dental bleaching: professional, which is performed by the dentist, and at-home, which the patient can do independently (12). Although the impact of HP on the color change of resin-based composites remains a topic of debate, it is generally agreed that various types of resin-based composites exhibit varying resistance to bleaching (15). While bleaching agents effectively remove extrinsic stains, they do not bleach composite materials to the same extent as natural tooth structure. Consequently, when a bleaching agent is applied, the color of the composite resin-based restoration may not always perfectly match that of the adjacent bleached tooth structure (16).

The objective of this *in vitro* study was to assess, using a spectrophotometer, the color stability of Class V restorations performed on anterior teeth with the universal composite Omnichroma before and after a professional teeth whitening.

## Material and Methods

The determination of the sample size was carried out using the statistical software G-Power v. 3.1 (University of Düsseldorf; Düsseldorf, Germany). A statistical significance analysis revealed that a sample size of 28 meets the constraints of α= 0.2 and power=0.90.

Twenty-eight dental elements from the anterior areas (central incisors, lateral incisors, and canines of both the upper and lower arches), extracted due to periodontal reasons or lost due to trauma, were collected and stored in a saline solution. They were carefully checked to select teeth that were free of caries, fractures and demineralization. The dental elements were cleaned of any remnants of periodontal ligament using curettes, rinsed with 10-volume hydrogen peroxide for 10 seconds, further rinsed with denatured alcohol for 30 seconds, and then stored in a saline solution to prevent dehydration.

The Class V cavities were prepared using a cylindrical diamond bur with a diameter of 1mm and a length of 5mm, following these parameters:

• A depth of 2mm, verified using a rubber stopper mounted on the bur ,

• Bucco-lingual cavity width of 3mm, measured with a periodontal probe,

• 0.5mm distance from the cementoenamel line, measured with a periodontal probe,

• 1mm distance from both the mesial and distal surfaces, measured with a periodontal probe.

The Class V restorations were then carried out following the protocol below:

• Etching with Tokuyama Etching Gel HV (Tokuyama Dental Corp, Japan): 30 seconds on enamel and 15 seconds on dentin,

• Adhesive procedure with Tokuyama EE Bond (Tokuyama Dental Corp, Japan) for 10 seconds on the entire cavity surface, removing the excess with air ,

• Polymerization for 20 seconds,

• Restoration with universal composite Omnichroma (Tokuyama Dental, Encinitas, CA, USA),

• Finishing and polishing with 2-step polishing system Enhance PoGo disks (Dentsply Caulk, Milford, DE, USA).

The teeth whitening was performed using Opalescence Boost (Ultradent Products, Inc.; USA), which is a 40% hydrogen peroxide-based gel, following the application protocol below:

• Drying of the sample,

• Activation of the whitening gel by mixing it with the activator by alternately pressing the plungers of the two syringes at least 25 times,

• Application of a layer of Opalescence Boost, approximately 1mm thick, on the vestibular surfaces of the tooth, left to act for 25 minutes,

• Aspiration of the whitening gel, rinsing with water, and drying with air,

• Procedure was performed twice.

The samples were stored in a saline solution.

The color measurements were performed by a single operator one week after the restoration (T1) and 24 hours (T2), 72 hours (T3), and 30 days after (T4) the teeth whitening procedure using the spectrophotometer MHT SpectroShade Micro® (2006, MHT Srl, Oxnard, CA, USA) as follows.

The dental elements were identified with an alphanumeric code to allow for color recordings at various time intervals and subsequent comparisons.

**Figure 1:**
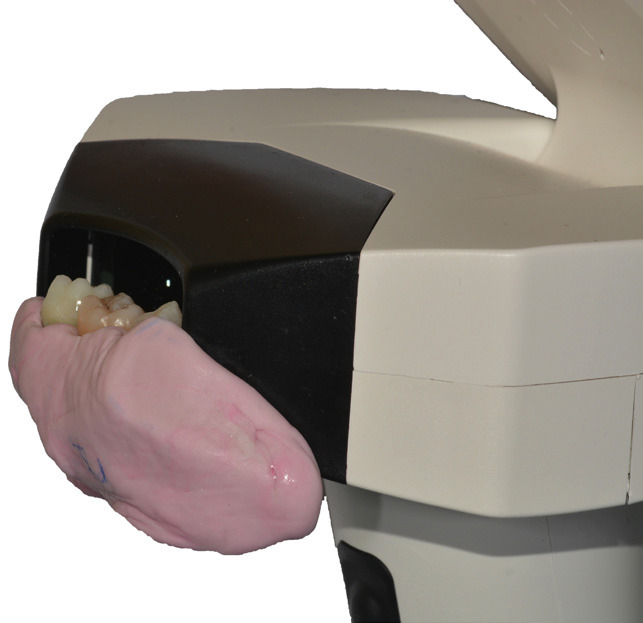
Silicone with dental elements positioned at the optical system of the spectophotometer.

**Figure 2:**
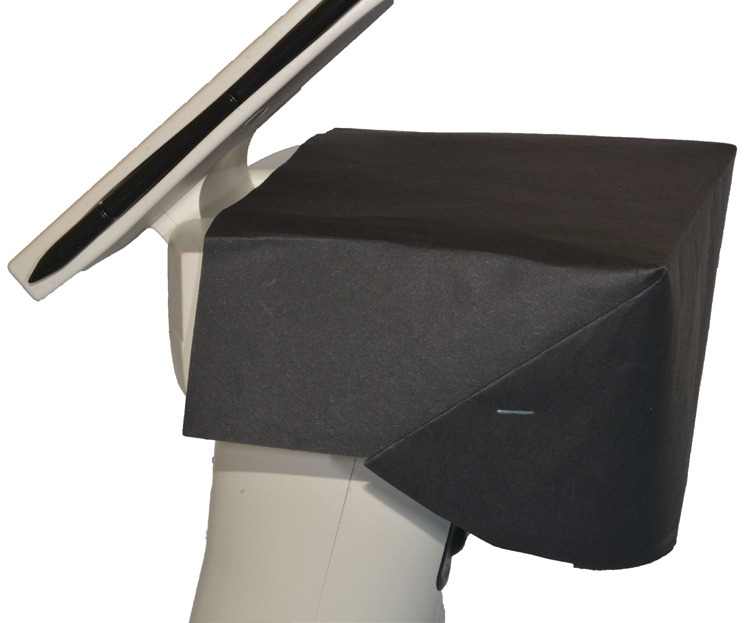
Black cardboard to simulate the low-light conditions of the oral cavity.

To record the color of each dental element, a support was created using pink condensation silicone (Zetalabor Putty Hard, Zhermack Dental, Germany) in order to simulate the gum tissue. Before the material hardened, three wells were created within the silicone. In the two lateral wells, two extracted premolars were inserted, used as reference points in all measurements to ensure standardized results. In the central well, the sample tooth to be analyzed was inserted. Once the silicone with the dental elements was positioned at the optical system of the spectrophotometer (Fig. 1), it was chosen to use a black cardboard to darken the area around the support in order to simulate the typical low-light conditions of the oral cavity (Fig. 2). Once captured, the images were downloaded onto a PC via the appropriate USB cable and were imported into the “MHT SpectroShade” software for storage.

The following parameters were evaluated:

1. L* parameter at T2 to assess the actual whitening of the dental element’s surface (identified as point P2).

2. L* parameter at T3 and T4 to check the long-term stability of the whitening effect on dental tissue (identified as point P2).

3. ΔE parameter obtained by analysing the differences in L*a*b* values between point P1 (restoration area) and point P2 (adjacent tooth area) to assess the chromatic difference between the dental surface and the restoration at different time intervals (T1 vs T2 vs T3 vs T4).

Using the MHT SpectroShade software to analyse the images at T1 for each dental element, two points, named P1 and P2, were selected and recorded. Point P1 was marked on the restoration area, while point P2 was marked on the surrounding dental surface. These two points were then reproduced in the same area in the images acquired at T2, T3, and T4. The circular selection tool provided by the software (size 20) was used to ensure that the dimensions of points P1 and P2 remained consistent in each recording.

The two coordinates (x and y) of each point on the dental element were identified and recorded using a digital ruler (ScreenRuler v.0.10.0, Bluegrams) to ensure that points P1 and P2 were always in the same area of tooth and restoration at each time interval.

**Figure 3:**
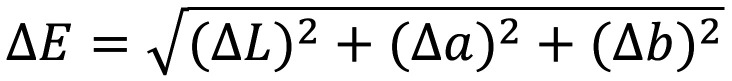
Formula.

The reference value of ΔE was calculated by comparing the L*a*b* values of P1 and P2 at each time interval as follows: (Fig. 3).

This approach allows for the assessment of the adaptation and chromatic stability of the restoration compared to the dental surface.

**Figure 4:**
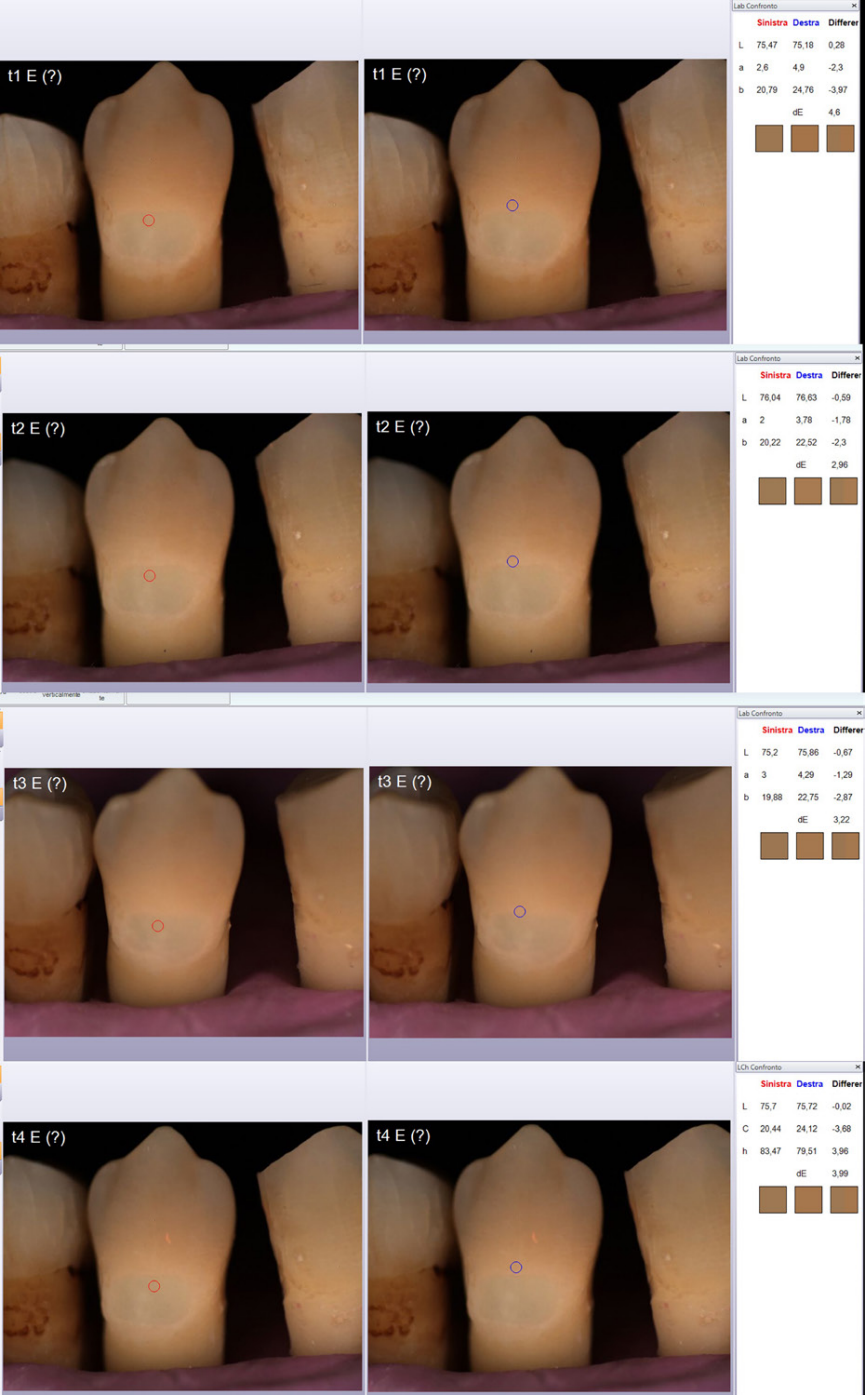
Images obtained at T1, T2, T3 and T4. Red circle indicates point P1 (restoration color reference), blue circle indicates point P2 (reference for the color of the surrounding tooth). In the right-hand column, the comparison between the L*a*b* values of points P1 and P2, as well as the value of ΔE, can be seen.

In the following image (Fig. 4), you can observe the images obtained at T1, T2, T3, and T4 of one of the twenty-eight samples in the study. The red circle indicates point P1 (restoration color reference), while the blue circle indicates point P2 (reference for the color of the surrounding tooth). In the right-hand column, the comparison between the L*a*b* values of points P1 and P2, as well as the value of ΔE, can be seen.

-Statistical Analysis

The data were analyzed using STATA16 statistical software (StataCorp, 1985, California USA) for the following purposes:

• Descriptive statistics (means and standard deviations) of L* and ΔE values at different time intervals of the experiment.

• The Shapiro-Wilk statistical test was used to check the normality of data distribution.

• To assess the actual whitening of the dental element, L* parameters at T1 and T2 were evaluated using the Wilcoxon-Mann-Whitney statistical test.

• To evaluate the stability of the whitening effect on dental tissue, L* parameters at T2, T3, and T4 were assessed using the Kruskal-Wallis statistical test.

• To assess the chromatic difference between the dental surface and the restoration (ΔE) at different time intervals (T1 vs T2 vs T3 vs T4), the non-parametric Kruskal-Wallis statistical test was employed.

A significance level of *P* ≤ 0.05 was used to indicate the statistical significance of the results.

## Results

The ΔE values for each dental element were collected and reported in  Table 1, obtained one week after the restoration with Omnichroma (T1) and 24 hours (T2), 72 hours (T3), and 30 days after the teeth whitening procedure (T4).

The descriptive statistics (mean and standard deviation) of the ΔE values at different time intervals are reported in  Table 2.

 Table 3 displays the L* values of point P2 (dental surface surrounding the restoration) for each sample obtained one week after the restoration with Omnichroma (T1) and 24 hours (T2), 72 hours (T3), and 30 days after the teeth whitening procedure (T4).

The descriptive statistics (mean and standard deviation) of these L* values are reported in  Table 4.

Descriptive statistics indicate that the highest L* value (74.28 (± 3.43)) for point P2 was obtained 24 hours after the whitening procedure (T2), while the lowest value (72.75 (± 4.06)) was recorded in the measurement taken one week after the restoration (T1).

Higher L* values indicate that the sample appears lighter, whereas lower L* values indicate that the sample appears darker.

3.1) Evaluation of the actual whitening of the dental element (L* parameter T1 vs. T2)

The Shapiro-Wilk test *p-value* for L* variables at T1 was 0.008. The *p-value* for L* variables at T2 was 0.06.

The non-parametric Wilcoxon-Mann-Whitney test resulted 0.11 indicating no statistically significant differences between the two variables.

The dental surface surrounding the restoration has undergone a clinical change in L* values before and after the dental whitening procedure (mean L* T1 < L* T2), however this change is not statistically significant.

3.2) Evaluation of the stability of the actual whitening of dental tissue (L* parameters T2 vs T3 vs T4)

The *p-value* of Shapiro-Wilk test for the L* variables at T2 was 0.06. The *p-value* for the L* variables at T3 was 0.02 and T4 was 0.01.

The non-parametric Kruskal-Wallis test resulted with a *p*=0.55 indicating no significant differences between the L* values at T2, T3, and T4. This clarifies that the color differences between the restoration and the tooth in terms of chromatic adaptation remain constant over time.

3.3) Evaluation of the chromatic difference between the dental surface and the restoration (ΔE) at different time intervals (T1 vs T2 vs T3 vs T4)

The *p-value* of the Shapiro-Wilk test for the ΔE variables at T1 was 0.25, T2 was 0.04, T3 was 0.06, and T4 0.24.

From the statistical analysis with the Kruskal-Wallis test, no statistically significant difference was found between the ΔE values at T1, T2, T3, and T4 (*p*=0.08), indicating that the ΔE value, at different timepoints, does not show statistically significant differences. Clinically, this result shows that the differences between the restoration and the tooth in terms of chromatic adaptation remain constant over time, indicating good material adaptability to whitening procedures.

## Discussion

The findings and their implications should be discussed in the broadest context possible. Future research directions may also be highlighted.

The objective of this *in vitro* study was to investigate the chromatic adaptability of restorations performed with the universal composite material Omnichroma in relation to the structure of the adjacent tooth before and after professional whitening procedures. The literature regarding this resin-based restorative material (Omnichroma) is currently limited, emphasizing the need for further research on this topic.

Clinicians have always desired composite materials and restoration techniques that allow for the adoption of simplified clinical protocols. This is important for reducing chair time and minimizing sensitivity to various operative techniques (10).

The most widely employed method for clinically assessing tooth color involves visual shade matching using a commercial shade guide (color scale). However, this method is generally considered inconsistent and subjective, as factors such as lighting, the surrounding environment, age, gender, eye fatigue, and color vision deficiencies can influence the visual selection of shades (17).

The difficulty in selecting the most suiTable composite shade for tooth surface has led to the development of so-called universal composites (10). Universal composite resins are designed to achieve an optimal aesthetic outcome since they offer a limited number of shades. Due to their enhanced “blending” effect with dental structure, they can naturally harmonize with the surrounding enamel and dentin. This effect, known as Color Adjustment Potential (CAP), enables composite resins to interact with adjacent dental tissues, minimizing color disparities (18).

In this research, the CIE L*a*b* color system was chosen to measure color differences between the restoration and the adjacent tooth structure using the SpectroShade Micro spectrophotometer (SpectroShade, Oxnard, CA, USA).

The threshold value for clinically accepTable ΔE values varies widely in the literature. When measuring ΔE, it becomes important to determine the human eye’s ability to detect a difference and establish whether such a difference is clinically significant. It would be crucial, therefore, to find concordance between ΔE values and the subjective perception of chromatic change. This proves to be a very important point to consider when aiming for an effective and useful translational outcome in color research.

Gross and Moser reported that AE* values between 0 and 2 represent imperceptible color differences, while ΔE values in the range of 2 to 3 represent just perceptible color differences (19).

Furthermore, it has been stated that a ΔE value of 3.3 represents the critical threshold for visual perception of color differences. This means that color changes with ΔE values greater than 3.3 are more likely to be visible to the average observer (20).

In conclusion, the ability to detect color variations can be considered a combination of human eye capabilities and operator expertise, and it is accepted that a ΔE value below 1.2 is predictably undetectable by the human eye. A ΔE between 1.2 and 3.3 is considered detectable by an experienced clinician, whereas a ΔE value greater than 3.3 is detectable by an untrained eye (21).

In accordance with the literature, values of ΔE below 3.3 were considered clinically acceptable in this study, meaning that mimicry is perceived favourably by the human eye below this threshold. Considering the mean ΔE values obtained at various time intervals (T1, T2, T3, T4), only the ΔE value obtained 24 hours after the whitening procedure (T2) was found to be clinically acceptable (ΔET2= 3.22 ± 1.32). The mean ΔE values obtained in the other time intervals, however, were not clinically acceptable (ΔET1= 4.06± 1.46; ΔET3= 3.69±1.34; ΔET4= 3.97±1.62). These results indicate that the chromatic difference between the tooth surface and the restoration (ΔE) slightly increases over time after whitening (ΔET3 > ΔET2 and ΔET4 > ΔET3), but it still remains lower than the difference recorded between the tooth and the restoration before the whitening procedures (ΔET1). This means that the color difference between the tooth and the restoration will become more noticeable one month after whitening, but it will still be less pronounced and more acceptable compared to the color difference between the tooth and the restoration before the whitening treatment. The obtained data can be justified by considering the effects of whitening agents on composite resins. Various hypotheses have been formulated in the literature regarding this matter. Firstly, the type of composite material, particularly the type of organic matrix, may play a role in how the color changes or stabilizes after whitening treatments. Additionally, the degree of conversion of the composite resin matrix into polymer can influence color stability because unconverted monomers could be susceptible to attack and degradation by whitening solutions (22) Other potential factors leading to color changes include the oxidation of surface pigments, which, being easily accessible, can make the restoration’s surface appear less intense in terms of color. Additionally, the oxidation of amino compounds has been identified as responsible for color instability over time (23).

In recent years, dissatisfaction with the color of teeth has been associated with an increased desire for treatments that enhance dental aesthetics. Tooth color can be improved through various methods and approaches, including whitening toothpaste, professional oral hygiene through scaling and polishing to remove stains and tartar, internal whitening of non-vital teeth, external whitening of vital teeth, enamel microabrasion using abrasives and acid, and the placement of crowns and veneers (24). Teeth whitening products typically help enhance the overall brightness of teeth by altering their intrinsic color and removing or controlling the formation of extrinsic stains. These products are usually based on hydrogen peroxide or carbamide peroxide, formulated in gels and applied to the teeth in various formats: through direct application, trays, or strips. Peroxide can penetrate inside the teeth, where it decolorizes or whitens the colored substances present in the tooth, giving them a whiter appearance (17). For this research, Opalescence Boost (Ultradent Products, Inc.; USA) was used, which is a hydrogen peroxide-based whitening product with a concentration of 40%, administered according to an in-chair protocol. Significant improvements in the color of dental hard tissues have been demonstrated following whitening procedures (25). Specifically, when considering the CIEL*a*b* color system, there is a significant increase in the L* color coordinate following a whitening procedure, while the a* and b* values are lower compared to the reference point. This indicates lighter and less yellow teeth after the treatment (26). It is evident that whitening agents (carbamide peroxide and hydrogen peroxide) used in teeth whitening can bring about noticeable color changes. At the same time, however, these products can cause alterations in the color and surface of resin-based composite restorations (27). Certainly, having a resin that can mimic the color of whitened teeth would result in patient satisfaction and a simultaneous avoidance of restoration replacements in such cases. There is currently a limited number of studies in the literature regarding the behaviour of universal composite resins, particularly Omnichroma composite, following dental whitening. In a recent study, Evans (2020) suggested that the shade difference between Omnichroma composite and the tooth decreases as the tooth becomes brighter, highlighting Omnichroma’s ability to adjust its shade after the surrounding tooth structure is whitened. The present study has achieved results that are in line with the aforementioned study. Indeed, the ΔE values between the Omnichroma restoration and the surrounding tooth surface at 24, 72 hours, and 30 days after the whitening procedure are lower than the ΔE values obtained before the whitening treatment. This would suggest that Omnichroma composite is capable of adapting to the color of the surrounding dental surface even after whitening procedures. However, despite the consistent results obtained in this study, some considerations about the research limitations are noteworthy. All the work was conducted *in vitro*; therefore, the results may differ from those *in vivo*. To mimic real clinical conditions, it would be interesting to subject the dental elements to the action of staining agents, such as tea or coffee, before performing the whitening procedures (28).

Another important consideration is the polishing and finishing protocol for the restorations themselves, where polishing can affect the pigmentation of the restoration resins, causing a change in shade that exceeds the acceptability threshold (29). The sample studied is limited to 28 dental elements and could be increased to obtain more consistent results. For example, the L* value obtained in the comparison between T1 and T2 was statistically non-significant, although there appeared to be a difference between the values at the two time points when looking at the means and standard deviations. Although a non-parametric test was used, we believe that the lack of statistical significance is attributable to the limited amount of data. The study is limited to the analysis of Class V restorations in anterior dental elements. It might be worthwhile to evaluate the same parameters on Class IV or Class III restorations, both because these types of restorations are more commonly encountered *in vivo* on anterior teeth and because the protocol for using Omnichroma in such restorations is different. In these cases, the protocol involves applying a thin layer of Omnichroma Blocker on the lingual wall beneath the Omnichroma composite to achieve an opacifying effect while preventing the passage of light. Only one teeth-whitening treatment method was analysed (professional whitening with Opalescence Boost using 40% hydrogen peroxide). Further studies should consider other concentrations of hydrogen peroxide gel, as they may have different effects on the degree of color change (22). It would also be interesting to evaluate different whitening methods, such as at-home treatment, where concentrations are lower but the contact time of the dental element with the whitening agent is prolonged.

Long-term effects were not documented within the scope of this study, and the current literature lacks information on follow-up. Several studies in the literature report follow-ups of up to 2 weeks. It would be interesting to subject the dental elements to thermal cycling to assess the effects of aging on the adaptability and color stability of restorations following whitening procedures (30).

## Conclusions

From the results, it appears that the color difference between the restoration and the adjacent tooth decreases following the whitening procedure. The color difference between the restoration and the adjacent tooth is clinically acceptable (ΔE<3.3) only at the 24-hour mark after whitening (T2). The universal composite Omnichroma has demonstrated its ability to adapt to the color of the surrounding tooth even after the surrounding tooth structure has been whitened. It is absolutely essential to establish a correlation between ΔE obtained through spectrophotometry and the perception of mimicry by the human eye, at least on average, in order to provide translational results of color rendering analysis.

## Figures and Tables

**Table 1:** ΔE values for each sample at T1, T2, T3 and T4.

id	ΔE T1	ΔE T2	ΔE T3	ΔE T4
A	3,55	2,52	3,18	3,12
B	6,52	6,42	6,32	6,33
C	3,66	2,15	2,26	3,74
D	3,99	2,69	3,25	6,66
E	4,6	2,96	3,22	3,99
F	2,66	1,98	2,57	1,96
G	5,43	4,65	5,72	6,53
H	5,61	2,46	2,67	4
I	3,2	4,25	4,27	3,97
J	3,42	2,65	3,47	3,43
K	6,41	4,54	6,21	7,55
L	4,5	2,78	3,38	4,14
M	2,89	3,05	3,78	3,24
N	3,93	4,91	4,52	4,46
O	3,71	3	3,49	3,8
P	2,12	1,61	1,8	1,92
Q	3,73	2,44	3,06	3,96
R	3,53	2,26	3,25	2,43
S	1,7	1,32	1,79	1,89
T	3,68	2,81	3,53	3,62
U	3,32	2,94	3,49	3,17
V	6,11	3,63	5,55	5,14
W	6,11	5,14	5,2	5,84
X	1,92	1,84	1,96	1,84
Y	4,48	3,07	2,8	2,59
Z	1,42	1,56	2,14	1,52
ALFA	6,02	5,47	4,67	4,79
BETA	5,41	4,93	5,74	5,64

**Table 2:** Mean ad standard deviations of ΔE at T1, T2, T3 e T4.

	Mean	SD
ΔE T1	4,06	1,46
ΔE T2	3,22	1,32
ΔE T3	3,69	1,34
ΔE T4	3,97	1,62

**Table 3:** L* values for each sample at T1, T2, T3 and T4.

id	L* T1	L* T2	L* T3	L* T4
A	70,03	71,83	72	72,13
B	69,58	70,46	70,5	70,18
C	70,52	71,57	73,73	72,92
D	71,63	72,49	70,65	69,94
E	75,18	76,63	75,86	75,72
F	78,27	79,25	78,91	78,57
G	72,08	73,81	74,27	73,17
H	60,75	66,5	66,48	65,35
I	74,6	75,11	75,27	74,76
J	74,19	73,92	75,14	75,28
K	71,43	73,9	71,7	71,55
L	71,92	74,08	71,61	71,92
M	74,11	75,14	75,08	74,62
N	73,62	74,38	74,25	74
O	72,95	75,05	74,13	73,5
P	77,3	77,74	77,17	77,4
Q	72,12	74,83	73,6	73,51
R	71,68	71,59	71,26	70,84
S	78,2	79,32	79,71	76,12
T	75,83	76,87	76,14	75,89
U	75	76,68	75,85	76,02
V	74,03	76,57	77,16	75,98
W	75,78	76,8	76,61	76,59
X	74,62	75,92	75,19	76,08
Y	74,13	77,83	75,37	74,96
Z	76,14	77,04	75,33	75,62
ALFA	65,07	68,42	65,33	66,7
BETA	64,88	66,13	65,24	65,56

**Table 4:** Mean and standard deviation of L* for each sample at T1, T2, T3 and T4.

	Mean	SD
L* T1	72,75	4,06
L* T2	74,28	3,43
L* T3	73,70	3,63
L* T4	73,39	3,41

## Data Availability

Not applicable.
